# Mechanisms Underlying Radioresistance and Reversal Strategies in Non-Small Cell Lung Cancer

**DOI:** 10.3390/ijms26146559

**Published:** 2025-07-08

**Authors:** Chenhui Zhao, Shilan Luo, Qing Shao, Peng Li, Litang Huang, Lu Meng, Hongxia Cheng, Anqi Zhang, Xiaomei Gong

**Affiliations:** Department of Radiation Oncology, Shanghai Pulmonary Hospital, Tongji University School of Medicine, Shanghai 200000, China; 2411319@tongji.edu.cn (C.Z.); luoshilan@tongji.edu.cn (S.L.); coyg_sq@163.com (Q.S.); 18701837650@126.com (P.L.); hltseu@163.com (L.H.); 2311259@tongji.edu.cn (L.M.); 20211210075@fudan.edu.cn (H.C.); anqizhang1014@163.com (A.Z.)

**Keywords:** non-small cell lung cancer, radiotherapy, radioresistance mechanisms, reversal strategies, potential biomarkers

## Abstract

Radiotherapy (RT) continues to be a fundamental component in the management of non-small cell lung cancer (NSCLC). Nevertheless, some NSCLC patients do not attain optimal therapeutic outcomes due to the emergence of radioresistance. Improving the effectiveness of RT in NSCLC necessitates a thorough comprehension of the mechanisms that lead to radioresistance. This review delineates various potential mechanisms of radioresistance in NSCLC, encompassing augmented DNA damage repair, cell cycle dysregulation, cancer stem cells (CSCs), epithelial–mesenchymal transition (EMT), tumor hypoxia, an immunosuppressive tumor microenvironment (TME), dysregulation of cell death pathways, metabolic reprogramming, exosome-mediated signaling, genetic mutations, aberrant activation of signaling pathways, and epigenetic modifications. In addition, this study explores various novel strategies aimed at enhancing the radiosensitivity of NSCLC and provides a concise overview of potential biomarkers predictive of RT response, which may contribute to the development of innovative combination therapies to address radioresistance and improve patient outcomes.

## 1. Introduction

Lung cancer continues to be the primary cause of cancer-related deaths globally [1]. NSCLC, accounting for approximately 80–85% of all lung cancer cases, encompasses subtypes including lung adenocarcinoma (LUAD), lung squamous cell carcinoma (LSCC), and large-cell carcinoma. For patients with stage I or II NSCLC, surgical resection remains the primary treatment modality. Therapeutic approaches for stage IIIB-IV NSCLC shift toward systemic therapies, including chemotherapy, targeted therapy, and immunotherapy, while RT is generally reserved for palliative care or oligometastatic disease [2]. NSCLC is often identified at an advanced stage, resulting in unfavorable clinical outcomes. Despite significant advancements in diagnostic and treatment methods, the five-year survival rate for people with NSCLC is still less than 20% [3].

RT entails the precise administration of high-energy radiation, such as X-rays, gamma rays, electron beams, protons, and charged particles, to eradicate tumor cells. Ionizing radiation (IR) causes biological damage either directly by disrupting cellular molecules or indirectly by generating free radicals during ionization processes [4]. Radiation therapy is frequently utilized alongside surgical procedures, chemotherapy, immunotherapy, or targeted treatments. RT is essential in the treatment of NSCLC, whether utilized independently or in conjunction with other modalities [5]. However, resistance to RT continues to be a significant obstacle to attaining optimal therapeutic efficacy.

Herein, this study investigates the mechanisms contributing to RT resistance in NSCLC, including DNA damage repair and cell cycle dysregulation, CSCs and EMT, hypoxia and immunosuppressive TME, abnormal regulation of cell death, metabolic dysregulation, exosomes, gene mutation, aberrant activation of pro-survival signaling pathways, and epigenetic dysregulation [6,7,8,9,10,11,12,13,14,15]. Mechanism-driven investigations provide critical insights that facilitate the systematic development of effective radiosensitizers.

In addition, we delineate contemporary strategies designed to surmount radioresistance, encompassing immunotherapy, targeted therapy, DNA damage repair modulation, hypoxia mitigation, cancer-specific metabolic pathways targeting, epigenetic regulation, and the advancement of nanotechnology-based radiosensitizers [16,17,18,19,20,21,22]. Our findings highlight potential biomarkers predictive of RT response, thereby laying a theoretical foundation for enhancing RT efficacy.

## 2. Mechanism of RT Resistance

### 2.1. DNA Damage Repair

The principal cytotoxic effect of radiation is facilitated by DNA damage, encompassing single-strand breaks (SSBs) and double-strand breaks (DSBs). Cancer cells repair IR-induced DNA damage through the activation of several DNA repair pathways, such as base excision repair (BER), homologous recombination (HR), and non-homologous end joining (NHEJ), thus circumventing cell death [23]. Mutations and modifications in DNA damage response (DDR)-related genes are commonly found in cancer and correlate with resistance to RT. Glycogen synthase kinase-3β (GSK-3β) increases the phosphorylation of DNA repair proteins, which aids in their recruitment to damage sites and enhances repair, ultimately contributing to resistance to RT [24]. Nicotinamide N-methyltransferase (NNMT) has been reported to be overexpressed in NSCLC. High levels of NNMT confer radioprotection by enhancing DNA repair through nicotinamide depletion [25]. NNMT silencing via siRNA significantly enhances radiosensitizing effects in 3D organoid models [26].

RT also produces reactive oxygen species (ROS) and reactive nitrogen species (RNS), resulting in oxidative cellular damage [27]. NSCLC cells frequently display increased concentrations of antioxidant proteins, which allow them to counteract ROS and mitigate IR-induced DNA damage, thereby reducing the efficacy of RT. Nuclear factor erythroid 2-related factor 2 (NRF2) promotes radioresistance primarily by enhancing the cellular antioxidant defense system, thereby reducing radiation-induced oxidative stress and DNA damage. Upon activation, NRF2 upregulates genes involved in detoxification, glutathione synthesis, and ROS scavenging, such as NQO1, HO-1, and GCLC. This protective environment promotes DNA repair processes, inhibits apoptosis, and maintains redox homeostasis. Additionally, NRF2-driven metabolic reprogramming increases NADPH production and sustains glutathione levels, further enhancing cell survival. These mechanisms enable cancer cells to better withstand ionizing radiation, thereby reducing the efficacy of RT [28,29].

### 2.2. Cell Cycle Dysregulation

The cell cycle consists of specific phases: the initial DNA synthesis phase (G1 phase), the DNA synthesis phase (S phase), the final DNA synthesis phase (G2 phase), and mitosis (M phase). The G1/S and G2/M checkpoints are essential for identifying and rectifying DNA DSBs. The activation of these checkpoints triggers cell cycle arrest, providing time for DNA repair and consequently enhancing tumor cell survival and resistance to RT. The EphrinA2 receptor (EphA2), part of the receptor tyrosine kinase family, is overexpressed in certain NSCLC cells. Its upregulation induces cell cycle arrest, diminishes sensitivity to RT, and amplifies NSCLC cell proliferation [30].

### 2.3. Cellular Senescence

Cellular senescence is a condition of irreversible cell cycle arrest frequently associated with the senescence-associated secretory phenotype (SASP), characterized by the release of pro-inflammatory cytokines, chemokines, angiogenic factors, and growth regulators. The SASP significantly influences tumorigenesis and contributes to radioresistance by affecting processes such as invasion, metastasis, EMT, and immune suppression [31]. Serine proteinase inhibitor E1 (PAI1), released by senescent tumor-associated mesenchymal stem cells (MSCs), has been recognized as an SASP factor that facilitates the progression of NSCLC and confers resistance to RT [32]. Lymphocyte immunoglobulin-like receptor B2 (LILRB2), recognized for its immunosuppressive characteristics, correlates with diminished RT responsiveness and unfavorable prognosis by promoting cellular senescence and enhancing SASP expression [33].

### 2.4. CSCs and EMT

CSCs are regarded as crucial factors in unfavorable prognosis and treatment resistance in NSCLC. The radioresistance of CSCs is linked to several intrinsic factors, such as increased DNA repair capability, modified ROS levels, regulation of the cell cycle, anti-apoptotic characteristics, and the activation of pro-survival signaling pathways. Moreover, extrinsic factors, especially the impact of the TME, significantly contribute to the modulation of CSC resistance to RT [34]. Heat shock 70-kDa protein 1-like (HSPA1L) facilitates CSC-like properties by modulating β-catenin transcription, consequently increasing radioresistance [35]. EMT endows tumor cells with enhanced invasive and migratory abilities and is linked to resistance against multiple anti-cancer treatments. In radiation-resistant NSCLC cells, the expression of EMT-related proteins is significantly increased. Tescalcin, which is significantly upregulated in NSCLC, augments EMT and CSC-like characteristics, thereby facilitating radioresistance [36]. IR has been demonstrated to promote stemness and EMT, consequently enhancing the motility and invasiveness of NSCLC cells [37].

### 2.5. Hypoxia and Immunosuppressive TME

**Figure 1:**
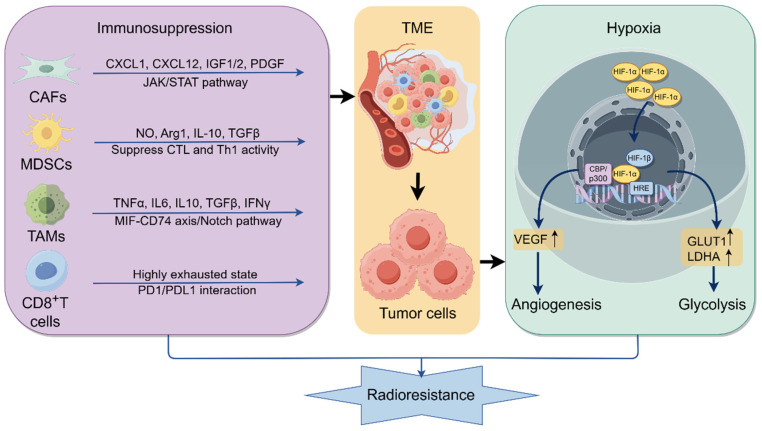
The hypoxic and immunosuppressive tumor microenvironment contributes to radioresistance in NSCLC. Hypoxia induces HIF-1α expression, which enhances the transcription of genes associated with angiogenesis and glycolysis via its interaction with hypoxia response elements in the nucleus, resulting in reduced radiosensitivity in NSCLC. Immunosuppressive cells, including CAFs, MDSCs, and TAMs, facilitate RT resistance by secreting various cytokines, activating intracellular signaling pathways, and interacting with other immune cells. Negative regulatory signals are conveyed to CD8^+^ T cells through the PD-1/PD-L1 axis, resulting in the inhibition of CD8^+^ T cells, the promotion of CD8^+^ T-cell apoptosis, and a significant reduction in immune response. (The upward arrow represents upregulation).

The TME is distinguished by hypoxia, acidity, and immunosuppression (Figure 1). Hypoxic tumors are typically more aggressive and exhibit reduced responsiveness to RT in comparison to well-oxygenated tumors. Hypoxia occurs in approximately 80% of NSCLC cases [38]. The enhanced radioresistance of hypoxic NSCLC cells is associated with reduced DNA damage and downregulation of DNA repair genes [10]. Hypoxic conditions enhance autophagy in tumor cells, thus facilitating resistance to RT [39]. Under hypoxic conditions, activated hypoxia inducible factor-1α (HIF-1α) translocates to the nucleus and binds to the hypoxia-responsive element. Elevated levels of HIF-1α drive the transcription of genes involved in VEGF signaling and glucose metabolism, thereby facilitating angiogenesis and glycolysis, which contribute to tumor invasion, metastasis, and resistance to RT [40,41]. Moreover, radiation therapy can increase HIF-1α expression by activating the PI3K/Akt/mTOR signaling pathway, which makes NSCLC cells more resistant to radiation [42]. Peroxiredoxin-1 (PRX-1), a transcriptional coactivator, enhances the DNA-binding activity of serum reactive factor. Radioresistant hypoxic lung cancer cells exhibit high expression levels of PRX-1. The PRX-1/TLR4 axis has been reported to promote hypoxia-induced RT resistance in NSCLC by targeting the NF-κB/p65 pathway [43].

Cancer-associated fibroblasts (CAFs) significantly contribute to resistance to RT by assisting tumor cells in evading radiation-induced apoptosis. CAFs facilitate radioresistance and tumor progression via the paracrine secretion of cytokines, including CXCL1, CXCL12, IGF1/2, and PDGF [44]. RT elicits diverse stress responses in the microenvironment, including the induction of senescence in CAFs. By triggering the JAK/STAT signaling pathway, these senescent-like CAFs improve proliferation and radioresistance in NSCLC [45]. Tumor-associated macrophages (TAMs) facilitate tumor proliferation and therapeutic resistance through the secretion of cytokines, activation of oncogenic pathways, and interaction with immune cells [11]. TAM infiltration is strongly linked to angiogenesis and lymphangiogenesis. Increased abundance of TAMs correlates significantly with poor overall survival (OS) in NSCLC patients (*p* = 0.023) [46]. It has been shown that the exosomal long non-coding RNA (lncRNA) AGAP2-AS1, which is released by M2-type macrophages, increases RT resistance in NSCLC by upregulating NOTCH2 expression and downregulating miRNA-296 [47].

An elevation in CD8^+^ T cells post-RT is an autonomous predictor of measurable tumor response [48]. Consequently, targeting fatigued CD8^+^ T cells offers a viable approach to surmounting RT resistance. CD39 is overexpressed in exhausted CD8^+^ T cell subsets. Combination of CD39 and RT reduces CD8^+^ T cell exhaustion and tumor growth [49]. Infiltration of myeloid-derived suppressor cells (MDSCs) exerts significant immunosuppressive effects and contributes to resistance to RT. The reduction of MDSCs, however, stimulates cytotoxic T lymphocyte (CTL) and T-helper 1 (Th1) responses in the TME, consequently increasing radiosensitivity in NSCLC [50]. Furthermore, NSCLC cells that endure RT frequently demonstrate increased PD-L1 expression, which enhances radiation resistance by enabling cell migration, inducing epithelial–mesenchymal transition, inhibiting apoptosis, and facilitating immune evasion [51].

### 2.6. Abnormal Regulation of Cell Death

Apoptosis, a meticulously regulated mechanism of programmed cell death (PCD), is frequently evaded by NSCLC cells to escape the cytotoxic effects of RT. These cells depend on ATM kinase to suppress pro-apoptotic signaling, thus enhancing survival during radiation stress [52]. The pancreatic progenitor cell differentiation and proliferation factor (PPDPF) enhances radioresistance by inhibiting the ubiquitination and degradation of the anti-apoptotic protein BABAM2, thereby maintaining its expression stability [53]. Moreover, apoptosis is dose-dependently promoted by the tumor suppressor protein p53. In NSCLC, its inactivation reduces RT sensitivity [34].

Autophagy, a catabolic mechanism that degrades and recycles cellular constituents, serves a dual function in cancer. In the initial phases, autophagy facilitates the removal of damaged cells and inhibits tumorigenesis. In advanced stages, autophagy facilitates tumor cell survival by preserving cellular homeostasis [54]. Aurora kinase A (AURKA) is a crucial regulator of autophagy, and the inhibition of the AURKA-CXCL5 axis triggers autophagic cell death, thereby increasing radiosensitivity in NSCLC [55]. Lactotransferrin (LTF), markedly upregulated in radioresistant NSCLC cells, facilitates autophagy and enhances resistance to RT [56].

Radiation therapy response is also linked to ferroptosis, a controlled form of cell death characterized by the accumulation of iron-dependent lipid peroxides. NSCLC cells that overexpress NRF2 demonstrate resistance to ferroptosis. Targeting NRF2 may mitigate RT resistance by facilitating ferroptosis and inducing mitochondrial dysfunction [57]. Pyroptosis, a recently discovered variant of programmed cell death, is facilitated by pyroptosis-associated proteins. In radioresistant NSCLC cells, the expression of R-spondin 3 (RSPO3) is markedly diminished. RSPO3 overexpression increases radiosensitivity by triggering pyroptosis through the activation of the NLRP3 inflammasome [58]. Mitotic catastrophe (MC), a specific type of cell death arising from aberrant mitosis, can be used to improve RT response. The p300 histone acetyltransferase inhibitor C646 enhances the sensitivity of NSCLC cells to RT by impairing G2 checkpoint regulation and inducing mitotic catastrophe [59].

### 2.7. Metabolic Dysregulation

**Figure 2:**
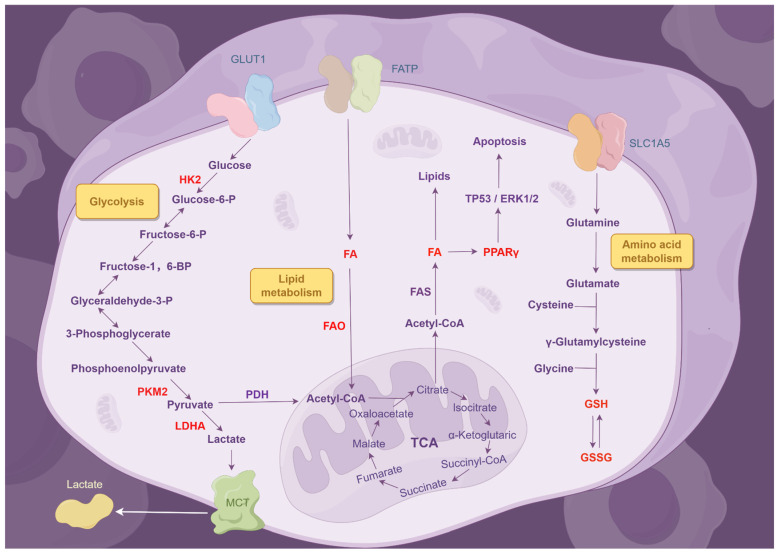
The correlation between metabolic reprogramming and radioresistance in NSCLC. Active glycolysis, amino acid metabolism, and lipid metabolism are fundamental traits of malignancy that enhance RT resistance in NSCLC by inhibiting tumor cell apoptosis and altering the tumor microenvironment.

Tumor cells often alter their metabolic pathways to meet the biosynthetic, energetic, and redox requirements of cancer (Figure 2). The Warburg effect, in which tumor cells favor glycolysis instead of oxidative phosphorylation despite the availability of oxygen, exemplifies significant metabolic reprogramming [60]. The aerobic glycolysis pathway is markedly upregulated in radiation-resistant NSCLC cells. It has been shown that pyruvate kinase M2 (PKM2), an essential regulator of glycolysis, can mediate this effect. Radiation-induced apoptosis and autophagy in NSCLC cells are enhanced when PKM2 expression is inhibited [61]. Mitochondrial dysfunction and related metabolic changes also contribute to radioresistance. Dichloroacetate (DCA) enhances mitochondrial activation by increasing pyruvate influx, leading to apoptosis through ROS production, and functions as an effective radiosensitizer in NSCLC [62].

Glutamine metabolism is crucial for maintaining redox balance and resistance mechanisms. Glutaminase catalyzes the conversion of glutamine to glutamate within the mitochondria, with glutamate subsequently acting as a precursor for the synthesis of glutathione (GSH). As an essential antioxidant, GSH preserves redox equilibrium. Selective inhibition of glutaminase enhances the radiosensitivity of NSCLC cells by depleting GSH, which intensifies radiation-induced DNA damage [63]. Moreover, the activation of the serine/glycine biosynthetic pathway enhances radioresistance by facilitating cell survival, proliferation, cancer stem cell maintenance, and redox homeostasis in NSCLC [64].

Radioresistant tumor cells demonstrate enhanced fatty acid (FA) synthesis and fatty acid oxidation (FAO) [65]. Changes in serum lipid and lipoprotein metabolism have been linked to unfavorable outcomes in NSCLC patients. Squalene epoxidase (SQLE), often overexpressed in malignancies, facilitates the transformation of squalene into oxidosqualene, an essential phase in cholesterol biosynthesis. SQLE inhibition impedes this pathway, resulting in squalene accumulation, endoplasmic reticulum (ER) stress, and increased radiosensitivity in NSCLC [66].

### 2.8. Exosomes

Exosomes are nanoscale membrane vesicles that range in size from 30 to 100 nanometers and are released into the extracellular environment. Exosomes are formed when multivesicular bodies fuse with the plasma membrane that surrounds them. These vesicles transport proteins, DNA, and RNA, among other biological materials, facilitating intercellular communication. Exosomes facilitate radioresistance by augmenting DNA repair processes, regulating apoptotic pathways, and altering the TME [13]. The hypoxic tumor microenvironment induces the secretion of stress-responsive factors, including heat shock protein 70 (HSP70). In addition to its intracellular role, HSP70 can be encapsulated in exosomes and released by NSCLC cells. There is a correlation between elevated levels of exosomal HSP70 and unfavorable clinical outcomes as well as increased radioresistance in NSCLC [67]. Angiopoietin-like 4 (ANGPTL4), a crucial modulator of angiogenesis, is secreted by hypoxic tumor cells and can be absorbed by normoxic adjacent cells, thereby inducing radiation resistance in these bystander cells by inhibiting ferroptosis and diminishing lipid peroxidation [68]. Exposure to high-energy IR induces significant DNA and cellular damage, resulting in changes to the cargo composition of exosomes originating from irradiated cells. These alterations influence intercellular communication and promote the spread of radioresistance [69]. Exosomal miR-208a, stimulated by X-ray irradiation, facilitates NSCLC cell proliferation and increases radioresistance by directly targeting the tumor suppressor p21 [70].

### 2.9. Gene Mutation and Aberrant Activation of Pro-Survival Signaling Pathways

Mutations in a number of oncogenes and tumor suppressor genes, including EGFR, KEAP1, KRAS, STK11, and ALK, characterize NSCLC. This overexpression of EGFR is seen in about 40–80% of NSCLC cases. EGFR mutations correlate with enhanced responses to RT and improved overall survival. EGFR inhibition increases radiosensitivity by triggering p53-dependent senescence in NSCLC cells [71]. Approximately 50% of local recurrences after RT occur in NSCLC tumors with KEAP1 mutations, underscoring its significance as a principal factor in radioresistance [72]. KRAS mutations, found in 25–30% of NSCLC cases, are associated with diminished RT efficacy and decreased patient survival [73]. Moreover, ALK translocations—deemed radiosensitive genotypes—exhibit enhanced local control relative to STK11-mutated or wild-type tumors [74].

The dysregulation of multiple signaling pathways is fundamental to the pathogenesis and resistance mechanisms of NSCLC. Increased NOTCH pathway activity is associated with poor prognosis and reduced radiosensitivity. Inhibiting NOTCH signaling mitigates radiation resistance by decreasing the population of tumor-initiating cells [75]. Activation of PI3K/AKT/mTOR correlates with local recurrence and poor disease-free survival (DFS) in patients with early-stage NSCLC undergoing stereotactic body radiation therapy (SBRT) [76]. Moreover, the misactivation of various signaling pathways—specifically Wnt/β-catenin, MAPK/ERK, HGF/c-Met, Hedgehog, Hippo, NF-κB, and JAK/STAT—is significantly associated with radioresistance in NSCLC. Moreover, IR can initiate pro-survival and proliferative pathways such as NF-κB, TGF-β, Wnt, Hedgehog, and NOTCH [77]. Innate immunity depends on the cGAS/STING pathway. In NSCLC, elevated levels of prostate cancer-associated transcript 1 (PCAT1) create an immunosuppressive milieu and enhance radioresistance by impeding T-cell activation via the suppression of the cGAS/STING signaling pathway [78].

### 2.10. Epigenetic Dysregulation

Epigenetic modifications, including DNA methylation, are essential in the regulation of gene expression. Aberrant DNA methylation, characteristic of numerous malignancies such as lung cancer, frequently entails hypermethylation of tumor suppressor genes and hypomethylation of oncogenes [79]. The expression of ovarian tumor family deubiquitinase 4 (OTUD4) is markedly diminished due to promoter hypermethylation, leading to improved DNA damage repair and heightened radioresistance in NSCLC patients [80].

Histone modifications, including acetylation, deacetylation, methylation, and demethylation, affect chromatin architecture and gene expression. Histone deacetylases (HDACs) inhibit gene expression by eliminating acetyl groups from histones. HDAC6 directly interacts with checkpoint kinase 1 (CHK1) and facilitates its ubiquitination. In HDAC6-knockdown NSCLC cells, CHK1 remains persistently active, resulting in G2 phase arrest, suppressed proliferation, and increased radiosensitivity [81]. Moreover, histone demethylation regulates chromatin dynamics and cellular functions. The histone demethylase inhibitor IOX1 enhances the sensitivity of NSCLC cells to RT by modifying chromatin accessibility and disrupting DNA repair mechanisms [82].

The ubiquitin–proteasome system (UPS) is crucial for protein degradation and homeostasis. The ubiquitin E3 ligase FBXO22 augments radiosensitivity by facilitating the degradation of PD-L1, thereby elevating DNA damage in NSCLC cells [83]. The absence of succinate dehydrogenase subunit 5 (SDH5) is correlated with enhanced RT outcomes; patients with diminished SDH5 expression demonstrate reduced tumor sizes one month following RT. The loss of SDH5 mechanistically inhibits p53 degradation through the UPS, resulting in enhanced apoptosis and increased radiosensitivity [84].

Non-coding RNAs (ncRNAs), devoid of protein-coding capability, modulate gene expression and influence critical cellular processes including proliferation, differentiation, invasion, and migration. By promoting β-catenin nuclear translocation and triggering the Wnt/β-catenin signaling pathway, the long non-coding RNA (lncRNA) LINC00921 increases resistance to RT [85]. MiRNA-384, markedly downregulated in NSCLC, augments radiosensitivity upon overexpression by diminishing G2/M cell cycle arrest, hindering DNA repair, and facilitating cell death [86]. In radioresistant NSCLC cell lines, circZNF208 is markedly upregulated. Its suppression enhances NSCLC cell sensitivity to X-ray radiation by regulating the miRNA-7-5p/SNCA signaling pathway [87].

## 3. Reversal Strategies for RT Resistance

In the management of most solid tumors, RT in conjunction with other therapeutic modalities has shown enhanced efficacy relative to monotherapy. For patients with locally advanced (stage III) NSCLC, the standard treatment involves the combination of RT and chemotherapy [88]. However, chemotherapy frequently entails considerable adverse effects, highlighting the pressing necessity for more efficacious and less toxic radiosensitizers. Despite comprehensive research, the majority of radiosensitizers for NSCLC are still in the experimental stage, with only a few advancing to clinical trials or practical use. The subsequent sections outline the existing strategies used to augment radiosensitivity in NSCLC (Table 1).

### 3.1. Immunotherapy for Radiosensitization

Immune checkpoint inhibitors (ICIs) reinstate antitumor immune responses by targeting checkpoint molecules, including programmed cell death protein 1 (PD-1) and its ligand, programmed death-ligand 1 (PD-L1). SBRT can induce the release of tumor-associated antigens (TAAs), facilitating dendritic cell (DC) maturation, cross-priming of CTLs, and increased lymphocyte infiltration into tumors, thus transforming immunologically “cold” tumors into “hot” ones [89]. In murine tumor models, the integration of RT with anti-PD-1 or anti-PD-L1 monoclonal antibodies induces significant CD8^+^ T cell responses, enhancing local tumor control, extending survival, and improving resistance to tumor rechallenge [90].

**Figure 3:**
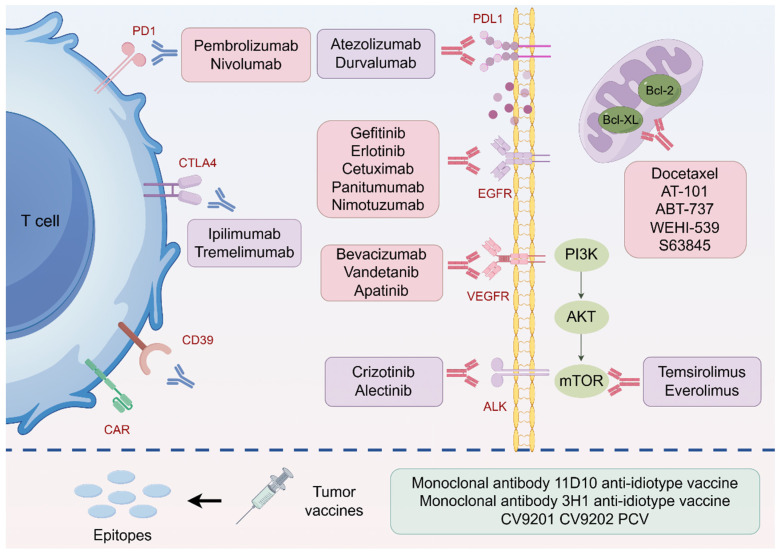
Potential therapeutic targets and agents to reverse NSCLC radioresistance. Combination strategies in ongoing investigations are presented, including combinations of multiple immunotherapies and targeted therapies.

Concurrent immunoradiotherapy has shown synergistic effects in impeding tumor progression and prolonging survival in NSCLC patients (Figure 3). A secondary analysis of the phase I KEYNOTE-001 trial (ID: NCT01295827) demonstrated that the PD-1 inhibitor pembrolizumab markedly enhanced OS in NSCLC patients with previous RT exposure, in contrast to those without, yielding a median OS of 10.7 months versus 5.3 months (*p* = 0.034) [91]. The multicenter, single-arm DOLPHIN phase II trial (ID: jRCT2080224763) demonstrated an objective response rate (ORR) of 90.9% (95% CI, 75.7%–98.1%) when the PD-L1 inhibitor durvalumab was combined with curative RT in patients with unresectable, PD-L1-positive, locally advanced NSCLC [92]. According to NCCN guidelines, dual checkpoint blockade utilizing PD-1/PD-L1 and cytotoxic T-lymphocyte-associated protein 4 (CTLA-4) inhibitors is endorsed as a category 1 treatment for metastatic NSCLC [93]. A phase Ib clinical trial assessing SBRT in conjunction with dual checkpoint inhibitors in 17 patients with oligometastatic NSCLC exhibited sustained progression-free survival (PFS), with a median PFS of 42 months, suggesting a possible therapeutic benefit compared to monotherapies [94].

Vaccines make the body build an immune response to kill tumor cells. Combining tumor vaccines and RT may lead to exceptional therapeutic outcomes. A subset of patients with ALK^+^ NSCLC may develop resistance to TKI treatment. The synergistic use of SBRT and pneumococcal conjugate vaccine (PCV) has shown complete pathological response (CPR) in a TKI-resistant patient [95]. The combined treatment can trigger immune responses and alter the immunosuppressive TME. Clinical trials have been evaluating the safety and tolerability of the tumor vaccines in combination with RT (ID: NCT00006470, NCT01915524).

### 3.2. Targeted Therapy for Radiosensitization

Epidermal growth factor receptor (EGFR) inhibitors comprise monoclonal antibodies (e.g., cetuximab) and small molecule tyrosine kinase inhibitors (TKIs) like erlotinib and gefitinib. The integration of RT with EGFR inhibitors improves local tumor management more efficiently than RT alone [96]. A phase II study of 252 NSCLC patients demonstrated significantly enhanced survival outcomes for those treated with erlotinib and RT, especially in patients with EGFR mutations [97]. Current clinical trials are assessing the effectiveness of EGFR-TKIs combined with RT in EGFR-mutant NSCLC (NCT01553942, NCT03521154). Bevacizumab, a monoclonal antibody that targets vascular endothelial growth factor (VEGF), has demonstrated encouraging outcomes. The REBECA phase I trial (ID: NCT01332929) exhibited enhanced response rates by integrating whole-brain radiotherapy (WBRT) with elevated doses of bevacizumab (15 mg/kg biweekly) in NSCLC patients with brain metastases (BM) [98].

Crizotinib, the inaugural ALK-TKI sanctioned for advanced ALK-positive NSCLC, markedly enhances prognosis when administered in conjunction with RT. In comparison to crizotinib monotherapy, the incorporation of RT elevated the overall response rate from 18% to 33% and enhanced progression-free survival in NSCLC patients with brain metastases to 7–27 months, in contrast to 3 to 4 months with crizotinib alone [99]. Preclinical studies indicate that mTOR pathway inhibition enhances the cytotoxicity of RT. A phase I trial (ID: NCT01396408) evaluating the combination of the mTOR inhibitor temsirolimus with thoracic RT indicated partial responses in 3 of 8 evaluable patients and stable disease in 2 patients, demonstrating good tolerability of the 15 mg weekly regimen [100].

In addition to targeting proliferation pathways, the promotion of apoptosis can augment the efficacy of RT. The Bcl-2 protein family, comprising Bcl-2, Mcl-1, Bak, and Bcl-xL, is frequently overexpressed in cancers owing to their anti-apoptotic properties. The pan-Bcl-2 inhibitor AT-101 increases radiosensitivity in NSCLC by facilitating apoptosis and diminishing proliferation in a concentration-dependent manner. Selective inhibitors of Bcl-xL (e.g., WEHI-539) and Mcl-1 (e.g., S63845) have exhibited radiosensitizing effects in preclinical models and are presently undergoing early clinical trials [12].

### 3.3. Modulating DNA Damage Repair

Poly (adenosine diphosphate-ribose) polymerases (PARPs) are essential enzymes that facilitate DNA damage repair. PARP inhibitors hinder the catalytic function of PARP proteins, consequently obstructing DNA repair processes. Preclinical data suggest that PARP inhibitors enhance radiation-induced DNA damage in NSCLC cells and impede tumor progression in irradiated xenograft models. A phase I trial (ID: NCT01562210) that combined radical RT with the PARP inhibitor olaparib demonstrated enhanced locoregional control and diminished treatment-related toxicity in patients with NSCLC [101].

The ATR-CHK1 complex facilitates DNA repair by initiating G2-M cell cycle arrest. M6620, a selective ATR kinase inhibitor, enhances the efficacy of RT in preclinical NSCLC models with brain metastases. MK-8776, a CHK1 inhibitor, increases the sensitivity of p53-deficient NSCLC tumors to RT by disrupting G2/M arrest and inhibiting double-strand break repair [102]. Numerous clinical trials have assessed ATR and CHK1 inhibitors in NSCLC, revealing encouraging therapeutic results (NCT02589522, NCT02873975, and NCT01115790) [103].

DNA-dependent protein kinase (DNA-PK) inhibitors impede catalytic function and inhibit DNA repair mechanisms. The DNA-PK inhibitor AZD7648 increases radiosensitivity by inducing genomic instability and facilitating apoptosis in NSCLC cells [7]. In xenograft models, M3814, in conjunction with a 6-week fractionated RT regimen, markedly improved the antitumor efficacy of RT and resulted in complete tumor regression at non-toxic dosages. M3814 is presently undergoing clinical evaluation in conjunction with RT (NCT02516813) [104].

Cyclin-dependent kinases (CDKs), specifically CDK4 and CDK6, govern the cell cycle at the G1 checkpoint in conjunction with D-type cyclins. The inhibition of CDK4/6 impedes the growth of NSCLC by triggering cell cycle arrest and apoptosis. Initial clinical trials and preclinical research have demonstrated that the combination of RT and CDK4/6 inhibitors produces tolerable toxicity and promising therapeutic efficacy across a range of tumor types [105].

### 3.4. Overcoming Hypoxia

Hypoxia is common in solid tumors and contributes to RT resistance in NSCLC by promoting cell proliferation, DNA repair, and cancer stemness. Strategies to address hypoxia in NSCLC encompass hypoxia molecular target inhibitors (such as HIF-1α inhibitors), hypoxia-activated prodrugs (including misonidazole and nimorazole), radiation sensitizers for hypoxic cells (like tirapazamine), agents that enhance oxygen delivery (for instance, efaproxiral), and medications that diminish oxygen consumption (such as metformin) [106].

Translational and clinical studies have demonstrated that the inhibition of HIF-1α diminishes antioxidant activity and tumor angiogenesis, modifies the TME, and increases radiosensitivity in solid tumors [19]. Misonidazole, a radiosensitizer characterized by high electron affinity, was previously evaluated in conjunction with RT for lung cancer but demonstrated dose-limiting neurotoxicity [106]. Tirapazamine selectively targets hypoxic tumor cells by inducing DNA strand breaks and base damage, thereby augmenting the efficacy of RT [107]. Efaproxiral diminishes hemoglobin’s affinity for oxygen, enhances oxygen release, and elevates tissue partial pressure of oxygen (pO_2_), demonstrating potential when used in conjunction with sequential chemoradiotherapy for NSCLC [108]. Additionally, the antidiabetic medication metformin augments RT response by inhibiting mitochondrial complex I, decreasing oxygen consumption, and enhancing tumor oxygenation [109].

### 3.5. Targeting Metabolic Processes

Targeting critical molecules implicated in glycolysis—namely glucose transporter 1 (GLUT1), hexokinase 2 (HK2), and lactate dehydrogenase A (LDHA)—can augment the radiosensitivity of NSCLC cells. Preclinical studies indicate that glycolysis inhibitors may enhance the effectiveness of RT by disrupting cellular redox balance and depleting ATP levels. The amalgamation of the HK2 inhibitor 2-deoxyglucose with hypofractionated RT has exhibited encouraging outcomes in multiple solid tumors during initial phase I/II clinical trials, with no notable toxicity reported [19].

The glutaminase inhibitor CB-839 increases radiosensitivity in NSCLC cells by depleting intracellular glutathione and hindering the scavenging of free radicals. Phase I clinical trials for patients with advanced solid tumors are currently being conducted on CB-839 [65]. Inhibition of serine/glycine metabolism impedes the post-RT recovery of cancer cells, resulting in enhanced tumor control in NSCLC. Notably, combining the serine/glycine conversion inhibitor sertraline with RT has been shown to enhance radiosensitivity and improve therapeutic efficacy in NSCLC patients (ID: NCT02921854) [64].

Fatty acid synthase (FASN), the primary enzyme in the de novo synthesis of long-chain fatty acids, is often upregulated in NSCLC and correlates with an unfavorable prognosis. Inhibiting FASN expression may increase radiosensitivity in NSCLC by diminishing the levels of DDR-related proteins and facilitating apoptosis [110]. FASN inhibitors, including epigallocatechin gallate (EGCG) and orlistat, have been employed as radiosensitizers to enhance RT outcomes [65].

### 3.6. Targeting Exosomes

Radioresistance facilitated by exosomes may be alleviated through the inhibition of exosome formation and release, or by altering their composition [69]. Amiloride, an inhibitor of sodium-calcium exchangers, can decrease exosome secretion and exhibit antitumor effects by inhibiting Stat3 phosphorylation and reducing the suppressive function of MDSCs in vivo [111]. Research is currently underway to clarify the function of exosomes in different subtypes of NSCLC. Exosomal PD-L1 has been linked to the effectiveness of immunotherapy in lung cancer patients [112], indicating that exosomes may represent tumor genetic background, burden, and treatment responsiveness. Moreover, engineered synthetic vesicles containing antitumor agents, gene-editing technologies, and immune-modulating molecules have shown promise in improving therapeutic precision, reducing toxicity, and inducing strong immune responses [113]. However, the clinical application of exosome-based radiosensitization strategies in NSCLC is still nascent and primarily confined to in vitro studies. Improvements in the stability and safety profiles of synthetic vesicles are essential for enhancing drug delivery accuracy and increasing radiosensitizing efficacy.

### 3.7. Regulating Epigenetics

The proliferation of NSCLC cell lines and xenograft models has been shown to be effectively inhibited by histone deacetylase inhibitors (HDACis). In conjunction with IR, HDAC inhibitors demonstrate significant synergistic effects across multiple cancer cell lines [15]. Vorinostat, an HDAC inhibitor, enhances radiation-induced cytotoxicity when used with RT and is well tolerated as both a monotherapy and in combination regimens. For NSCLC patients with brain metastases (BM), a phase I clinical trial conducted by Choi CYH et al. determined that 400 mg/day of vorinostat combined with stereotactic radiosurgery (SRS) was the maximum tolerated dose [114]. Aberrant DNA methylation, chiefly facilitated by DNA methyltransferases (DNMTs), contributes to radioresistance. The DNMT inhibitor 5-aza-2’-deoxycytidine facilitates the demethylation of tumor suppressor gene promoters, such as p53 and p21, consequently reinstating their expression. This reactivation amplifies apoptotic responses and suppresses oncogenic signaling post-RT, leading to increased radiosensitivity [21].

### 3.8. Nanoradiosensitizers

Nanomaterials have emerged as effective radiosensitizers owing to their ability to absorb, scatter, and emit radiation, alongside their chemical stability, biocompatibility, and minimal systemic toxicity. Radiosensitizing agents, such as chemotherapeutics, oxygen carriers, siRNAs, and catalases, can be encapsulated in hollow nanoshells for targeted delivery to tumor locations [115]. Gold nanoparticles, due to their extensive surface area, augment the generation of ROS and oxidative stress, consequently intensifying radiation effects. Furthermore, gold nanoparticles can enhance radiosensitivity by directly engaging with DNA and modifying cell cycle progression [116]. Cerium oxide nanozymes (CeO_2_), encapsulated and administered through zeolitic imidazolate framework-8 (ZIF-8), demonstrate significant catalytic efficacy in the conversion of hydrogen peroxide (H_2_O_2_) to oxygen. The CeO_2_@ZIF-8-HA complex effectively alleviates tumor hypoxia and improves radiation response in NSCLC [117]. Moreover, Nanosystems have proven effective in delivering radioactive seeds, including ^225^Ac (which emits α-particles), ^131^I, and ^125^I, directly to tumor locations, facilitating precise and efficient brachytherapy.

Although existing radiosensitization strategies have demonstrated efficacy in certain patients, they continue to face significant limitations. For example, while PARP inhibitors enhance radiosensitivity by impairing DNA damage repair, their effects are not tumor-specific and may compromise DNA repair capacity in normal tissues, leading to severe hematologic toxicity. Under specific conditions, RT may paradoxically diminish the efficacy of immunotherapy, especially when radiation fields encompass lymphoid organs, as this can deplete T-cell populations essential for immune responses. Furthermore, RT-induced PD-L1 upregulation, although providing a target for immunotherapy, concurrently amplifies tumor immune evasion. Most current clinical trial designs predominantly rely on empirical treatment protocols that lack biomarker-guided patient stratification, often resulting in suboptimal therapeutic outcomes. With rapid advancement in molecular diagnostics, biomarker-driven precision RT strategies now enable accurate patient stratification and personalized treatment planning through the identification of predictive markers for radiosensitivity.

## 4. Predictive Biomarkers of Radiosensitivity

Strategically targeting molecular determinants that affect RT sensitivity offers potential for surmounting radioresistance. This context presents a thorough review of potential novel biomarkers linked to radiosensitivity in NSCLC (Table 2). Despite their promise in personalizing RT, radiosensitivity biomarkers are subject to certain limitations, including biological complexity, technical variability, and inadequate clinical validation. For example, radiosensitivity is influenced by multiple factors, such as DNA repair capacity, hypoxia, tumor metabolism, and immune contexture. However, most biomarkers have been evaluated in isolation, without consideration of interactions among these pathways. To overcome these limitations, future research should prioritize the development of integrated biomarker panels, AI-driven predictive modeling, and prospective clinical trials.

## 5. Conclusions and Perspectives

Recent technological advancements have markedly enhanced the accuracy and effectiveness of RT. However, a segment of NSCLC patients persists in having inadequate treatment results due to intrinsic or acquired resistance to RT. Multiple preclinical and clinical studies yield valuable insights for developing effective strategies to reverse RT resistance. Due to the intricate and diverse nature of resistance mechanisms, it is advisable to conduct gene sequencing before RT in NSCLC patients to detect pertinent mutations, along with aberrantly expressed genes, proteins, and signaling pathways. Throughout RT, innovative pharmaceutical agents that target radioresistance-related pathways—such as those implicated in metabolism, EMT, cGAS/STING signaling, and DDR—may function as immunopotentiators to improve therapeutic results. Gene-editing technologies, especially CRISPR/Cas9, present promising opportunities for tackling genetic factors of resistance. Moreover, the development of sophisticated radiosensitizers and the identification of novel therapeutic targets can benefit from artificial intelligence technologies. The formulation of radiosensitization regimens must be accompanied by a comprehensive assessment of toxicity profiles to guarantee the safety and tolerability of combination therapies.

## Figures and Tables

**Table 1:** Clinical trial of radiosensitizers combined with RT for patients with NSCLC.

Mechanisms	Targets	Radiosensitizers	Phases	Primary Endpoints	Registration
Targeting immune checkpoints	PD1	Pembrolizumab	Phase I	ORR	NCT01295827
	PD1	Pembrolizumab	Phase II	OS, PFS	NCT02343952
	PDL1	Durvalumab	Phase II	PFS	jRCT2080224763
	CTLA4	Ipilimumab	Phase II	OS, PFS	NCT02221739
	PD1 + CTLA4	Nivolumab + Ipilimumab	Phase I	ORR, PFS	NCT03223155
	PDL1 + CTLA4	Durvalumab + Tremelimumab	Phase II	OS, PFS	NCT05000710
Tumor vaccines	Tumor antigens	Monoclonal antibody 11D10 anti-idiotype vaccine and monoclonal antibody 3H1 anti-idiotype vaccine	Phase II	OS, PFS	NCT00006470
	Tumor antigens	CV9202	Phase I	OS, PFS	NCT01915524
Targeting cell proliferation	EGFR	Osimertinib	Phase III	OS	NCT03521154
	EGFR	Gefitinib	Phase I	ORR, PFS	NCT00328562
	EGFR	Erlotinib	Phase II	OS, PFS	NCT00563784
	EGFR	Cetuximab	Phase III	ORR	NCT00115518
	EGFR	Panitumumab	Phase II	OS	NCT00979212
	EGFR	Nimotuzumab	Phase II	OS, PFS	NCT00872482
	ALK	Ceritinib	Phase II	OS, PFS	NCT02336451
	ALK	Crizotinib	Phase II	OS, PFS	NCT02314364
	ALK	Alectinib	Phase I/II	PFS	NCT05724004
	PI3K	Buparlisib	Phase I	MTD	NCT02128724
	mTOR	Temsirolimus	Phase III	MTD	NCT00796796
	mTOR	Everolimus	Phase I	MTD	NCT01063478
	PKC	Enzastaurin	Phase II	PFS	NCT00415363
Targeting angiogenesis	VEGF	Endostar	Phase II	OS, PFS	NCT01733589
	VEGF	Bevacizumab	Phase II	PFS	NCT04345146
	VEGF	Vandetanib	Phase I	MTD	NCT00807170
	VEGFR	Anlotinib	Phase II	PFS	NCT03672136
Targeting cell apoptosis	Bcl-2/Bcl-xL	Docetaxel	Phase I	MTD	NCT00378404
Targeting DNA damage repair	PARP	Olaparib	Phase I	ORR	NCT01562210
	PARP	Veliparib	Phase II	OS, ORR	NCT01657799
	ATR	Berzosertib	Phase I	PFS	NCT02589522
	ATR	Ceralasertib	Phase I	MTD	NCT04550104
	CHK1	Prexasertib	Phase II	OS, PFS	NCT02873975
	DNA-PK	M3814	Phase I	ORR	NCT02516813
Targeting hypoxia	HIF-1α	Nitroglycerin	Phase II	ORR	NCT06238882
	HIF-1α	Topotecan	Phase I	MTD	NCT00002537
	Top II	Tirapazamine	Phase I	ORR	NCT00033410
	Hb	Efaproxiral	Phase III	OS, PFS	NCT00055887
	Mitochondrial complex I	Metformin	Phase II	OS, PFS	NCT02186847
Targeting inflammation	COX-2	Celecoxib	Phase II	ORR	NCT00181532
Targeting metabolism	Glutaminase	CB-839	Phase I	ORR	NCT02071862
Targeting epigenetics	HADC	Vorinostat	Phase I	MTD	NCT00946673
	Proteasome	Bortezomib	Phase I/II	OS	NCT00093756

**Table 2:** Potential novel strategies and biomarkers under investigation in preclinical studies.

Mechanisms	Targets	Drugs	Conclusion	Reference
Immunosuppressive TME	CD39	CD39i	Inhibition of CD39 combined with RT preferentially decreases the percentage of exhausted CD8^+^ T cells.	[49]
	TAMs	Clodronate	Depletion of TAM by clodronate was sufficient to abrogate aerobic glycolysis and tumor hypoxia, thereby improving tumor response to anticancer therapies.	[118]
DNA damage repair	CHK1	MK-8776	MK-8776 radiosensitized p53-defective NSCLC by abrogation of G2/M arrest and by inhibition of DSB repair.	[102]
	ATM	BIBR1532	BIBR1532 enhances radiosensitivity of NSCLC through increasing telomere dysfunction and ATM/CHK1 inhibition	[119]
	DNA-PK	AZD7648	AZD7648 is an efficient sensitizer of radiation-induced DNA damage.	[7]
	DNA-PK	Ku-DBi	Ku-DBis inhibit cellular DNA-PK, NHEJ-catalyzed DSB repair and sensitize NSCLC cells to DSB-inducing agents.	[120]
	NNMT	Macrocyclic peptides, GYZ-319	Macrocyclic peptides and GYZ-319 show potent inhibitory effects against NNMT.	[121,122]
Cell cycle dysregula-tion	CDK4/6	Abemaciclib	Abemaciclib combined with IR increases radiosensitivity in NSCLC in preclinical models.	[105]
	CDK4/6	Palbociclib	Palbociclib in combination with MEK inhibitor has significant anti-NSCLC activity and radiosensitizing effect in preclinical models.	[123]
Hypoxia	GPX	Misonidazole	Misonidazole exhibits radiosensitizing effects in human LSCC.	[106]
Abnormal regulation of cell death	Bcl-2	AT-101	AT-101 inhibits Bcl-2 and leads to radiosensitization of NSCLC.	[124]
	Bcl-2	ABT-737	Combined inhibition of Bcl-2 and mTOR amplifies radiosensitization in NSCLC xenografts by simultaneously inducing apoptosis and autophagy.	[125]
	Mcl-1/Bcl-xL	WEHI-539/ S63845	Inhibition of Mcl-1 and Bcl-xL can result in increased radiation-induced cytotoxicity in NSCLC cell lines.	[12]
	cIAP1/2	Birinapant	Birinapant-induced apoptosis and inhibited the proliferation of NSCLC cells after exposure to radiation.	[126]
Metabolic dysregulation	Serine/glycine	Sertraline	The combination of sertraline and RT diminished the proliferation, clonogenicity, and self-renewal capacity of NSCLC stem cells.	[64]
	SQLE	Terbinafine	SQLE inhibition increases radiation efficacy in NSCLC by impairing cholesterol synthesis and increasing squalene levels.	[66]
	Pyruvate	Dichloroacetate	Dichloroacetate radiosensitizes NSCLC by increasing the influx of pyruvate and promoting mitochondrial activation.	[62]
Epigenetic dysregulation	DNMT	5-aza-2’-deoxycytidine	5-aza-2’-deoxycytidine promotes radiosensitivity by enhancing apoptosis and blocking oncogenic signaling.	[21]
	Histone demethylase	IOX1	IOX1-mediated inhibition of demethylase alters chromatin accessibility, thereby increasing radiation sensitivity in NSCLC.	[82]
	Histone demethylase	PBIT	The H3K4me3 demethylase inhibitor PBIT enhances the sensitivity of cancer cells to radiation.	[127]
Nanoradiosensitizers	CD44	Mn-Zn ferrite magnetic nanoparticles	Improving targeted cancer therapy through the integration of hyperthermia and RT utilizing Mn-Zn ferrite magnetic nanoparticles.	[128]
	Hydroxyl radical	GONs	GONs augment hydroxyl radical generation and cellular damage during carbon ion irradiation.	[129]
	-	AuNP	AuNPs enhance radiation effects via physical, chemical and biological interactions with IR.	[116]
	QT/CeO_2_	CeO_2_@ZIF-8-HA nanoparticles	The nanocomplexes catalyze the decomposition of H_2_O_2_ into O_2_, thereby markedly alleviating the hypoxia of the tumor microenvironment, while the radiosensitizer QT induces direct DNA damage post-radiotherapy.	[117]

## Data Availability

The original contributions presented in this study are included in the article. Further inquiries can be directed to the corresponding author.
